# Integrated use of plant growth-promoting bacteria and nano-zinc foliar spray is a sustainable approach for wheat biofortification, yield, and zinc use efficiency

**DOI:** 10.3389/fpls.2023.1146808

**Published:** 2023-05-08

**Authors:** Arshad Jalal, Carlos Eduardo da Silva Oliveira, Guilherme Carlos Fernandes, Edson Cabral da Silva, Kaway Nunes da Costa, Jeferson Silva de Souza, Gabriel da Silva Leite, Antonio Leonardo Campos Biagini, Fernando Shintate Galindo, Marcelo Carvalho Minhoto Teixeira Filho

**Affiliations:** ^1^ Department of Rural Engineering, Plant Health and Soils, São Paulo State University (UNESP), Ilha Solteira, Brazil; ^2^ Faculty of Agricultural Sciences and Technology, Department of Plant Production, São Paulo State University (UNESP), Dracena, Brazil

**Keywords:** *Triticum aestivum L.*, zinc fertilization, beneficial microorganisms, *Azospirillum brasilense*, *Bacillus subtilis*, *Pseudomonas fluorescens*, PGPBs

## Abstract

**Introduction and aims:**

The intensive cropping system and imbalance use of chemical fertilizers to pursue high grain production and feed the fast-growing global population has disturbed agricultural sustainability and nutritional security. Understanding micronutrient fertilizer management especially zinc (Zn) through foliar application is a crucial agronomic approach that could improve agronomic biofortification of staple grain crops. The use of plant growth-promoting bacteria (PGPBs) is considered as one of the sustainable and safe strategies that could improve nutrient acquisition and uptake in edible tissues of wheat to combat Zn malnutrition and hidden hunger in humans. Therefore, the objective of this study was to evaluate the best-performing PGPB inoculants in combination with nano-Zn foliar application on the growth, grain yield, and concentration of Zn in shoots and grains, Zn use efficiencies, and estimated Zn intake under wheat cultivation in the tropical savannah of Brazil.

**Methods:**

The treatments consisted of four PGPB inoculations (without inoculation, *Azospirillum brasilense, Bacillus subtilis*, and *Pseudomonas fluorescens*, applied by seeds) and five Zn doses (0, 0.75, 1.5, 3, and 6 kg ha^−1^, applied from nano ZnO in two splits by leaf).

**Results:**

Inoculation of *B. subtilis* and *P. fluorescens* in combination with 1.5 kg ha^−1^ foliar nano-Zn fertilization increased the concentration of Zn, nitrogen, and phosphorus in the shoot and grain of wheat in the 2019 and 2020 cropping seasons. Shoot dry matter was increased by 5.3% and 5.4% with the inoculation of *P. fluorescens*, which was statistically not different from the treatments with inoculation of *B. subtilis* as compared to control. The grain yield of wheat was increased with increasing nano-Zn foliar application up to 5 kg Zn ha^−1^ with the inoculation of *A. brasilense* in 2019, and foliar nano-Zn up to a dose of 1.5 kg ha^−1^ along with the inoculation of *P. fluorescens* in the 2020 cropping season. The zinc partitioning index was increased with increasing nano Zn application up to 3 kg ha^−1^ along with the inoculation of *P. fluorescens*. Zinc use efficiency and applied Zn recovery were improved at low doses of nano-Zn application in combination with the inoculation of *A. brasilense, B. subtilis*, and *P. fluorescens*, respectively, as compared to control.

**Discussion:**

Therefore, inoculation with *B. subtilis* and *P. fluorescens* along with foliar nano-Zn application is considered a sustainable and environmentally safe strategy to increase nutrition, growth, productivity, and Zn biofortification of wheat in tropical savannah.

## 1 Introduction

Zinc (Zn) malnutrition and deficiency is a persistent health and social concern that has affected approximately 17.5% of the global population (Bollinedi et al., 2020; Maxfield et al., 2023). Zinc deficiency is recognized as the 11th major health risk factor in the world and 5th in developing countries, and is declared as a “hidden hunger” (Bhatt et al., 2020; Poniedziałek et al., 2020). The high phytate–Zn ratio is another factor that can hinder Zn distribution into the edible tissues of cereal crops (Rehman et al., 2021). The prevalence of Zn malnutrition is most commonly seen in the population of wheat-consuming countries due to its cultivation on marginal and Zn-deficient soils (Chattha et al., 2017). In addition, several other factors including soil pH, bicarbonates, oxides, macronutrient concentration, and low mobility of Zn in soil solution affect Zn use efficiency and availability to the plants that can lead to crop failure, inadequate Zn accumulation in edible tissues, and human malnutrition (Bhatt et al., 2020; Zulfiqar et al., 2021; Penn et al., 2023). Therefore, to improve the quality and productivity of field crops, an alternative potential source of fertilizers is needed to replace conventional Zn fertilizers.

In recent years, the use of nano-Zn fertilizer is considered as an effective tool in the agricultural system because of its multiple impacts on plants and the environment (Aziz et al., 2019; Ahmad et al., 2020). The application of nano-Zn fertilizer can increase Zn mobility in phloem, increasing its bioavailability in the endosperm and contributing to protein synthesis and other biochemical traits of crop plants (Rossi et al., 2019; Ahmad et al., 2020). In addition, foliar nano-Zn application is considered as a less expensive and rapid strategy in comparison to soil Zn fertilization for better performance and agronomic biofortification of crop plants under different environmental conditions (Faizan et al., 2021; Jalal et al., 2022c). The application of nano-Zn in the early or late growth stages of crops can better define the effectiveness of agronomic biofortification under field conditions (Afshar et al., 2020; Jalal et al., 2022c). Nano-Zn application during tasseling and grain filling growth stages of wheat and maize could improve grain development and grain Zn concentration under field conditions (Jalal et al., 2022c; Jalal et al., 2023). However, foliar fertilization is limited by source, particulate size, and formulation (Fernández and Brown, 2013), which may cause toxicity in field crops and humans. Adopting sustainable agricultural practices to face the challenge of food and nutritional security in a sustainable manner is unprecedented.

The intervention of plant growth-promoting bacteria (PGPBs) in an agricultural system is a well-known sustainable strategy that could improve soil fertility, crop productivity, and nutrient bioavailability to deal with food and nutritional security (Akbar et al., 2019; Jalal et al., 2020c; Kumar et al., 2021). PGPBs can enhance Zn bioavailability and accumulation through solubilization, nitrogen fixation, synthesis of inorganic and organic acids, phyto-hormones, and chelators (Idayu et al., 2017; Khoshru et al., 2020). Inoculation with PGPBs could induce plant growth and performance by increasing nutrient use efficiency, improving water retention and synthesis of secondary metabolites, and protecting host plants against biotic and abiotic stresses (Jalal et al., 2021; Hungria et al., 2022; Ilyas et al., 2022; Yasmin et al., 2022). Among PGPBs, *Azospirillum brasilense, Bacillus subtilis*, and *Pseudomonas fluorescens* are the most studied inoculants in Brazil. The combined Zn fertilization and inoculation with *A. brasilense* can boost the productivity of tropical cereal crops by enhancing Zn absorption and use efficiency (Galindo et al., 2021). Additionally, *B. subtilis* and *P. fluorescens* are recently identified as the most effective inoculants for solubilizing Zn and phosphorus as well as for enhancing plant growth and performance under diverse environments (Ahmad et al., 2021; Jalal et al., 2021; Jalal et al., 2022a; Jalal et al., 2022b; Rosa et al., 2022).

Wheat has been recognized as a delicate cereal crop due to its naturally low grain Zn concentration (Zou et al., 2012). Wheat has a great agronomic relevance to food and nutritional security and its cultivation in tropical savannah can be considered as a promising approach towards achieving food security and sustainability (FAO. United Nations Food and Agricultural Organization, 2019; Galindo et al., 2022a). Wheat cultivation in tropical and marginal regions can be a diversified source of food to feed the increasing global population with optimal nutritional food (Galindo et al., 2022a). Therefore, inoculation with PGPBs in combination with Zn fertilizer could be considered as a sustainable strategy to improve nutrient use efficiency and wheat productivity (Galindo et al., 2021). However, there still exists a research gap on the combined application of PGPBs and foliar fertilizer of nano-Zn on the Zn use efficiency, nutritional status, and yield of wheat in tropical savannah. Hence, the present study hypothesized that the combined use of PGPBs and foliar fertilizer of nano-Zn would improve Zn use efficiencies and was assumed to be a sustainable strategy for increasing wheat productivity and biofortification. In the present scenario, the aim of the current study was to identify the best-performing PGPB inoculant in combination with nano-Zn foliar application on wheat growth, grain yield, biofortification, Zn use efficiencies, and estimated daily Zn intake in the tropical savannah of Brazil.

## 2 Materials and methods

### 2.1 Experimental area and location

**Figure 1:**
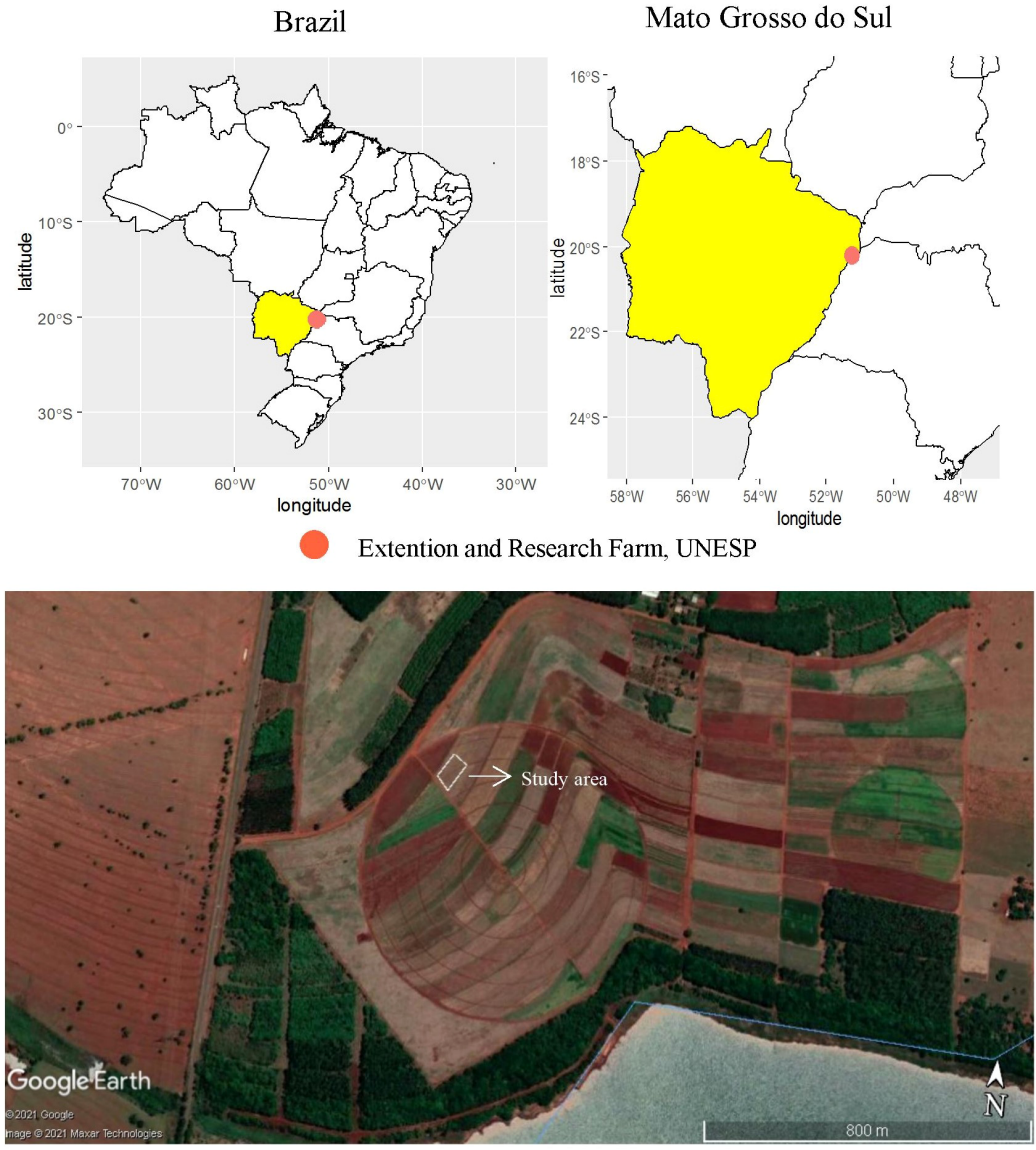
Experimental location at the Research and Extension Farm in Selvíria - Mato Grosso do Sul state, Brazil (20°22′S, 51°22′W, altitude of 335 m) in 2019 and 2020 crop seasons. The map was created using pacot, geobr, and ggplot with R software (R Development Core Team, 2015). Projection System WGS 84/UTM 200DC [EPSG: 4326]. This image was taken from Google Earth program, Google Company (2021). Map data: Google, Maxar Technologies.

**Figure 2:**
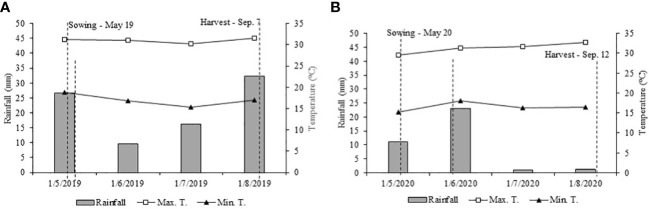
Meteorological data were acquired from the automatic weather station of Education and Research Farm during the wheat cultivation period from May to September 2019 **(A)** and May to September 2020 **(B)**.

A field experiment was performed with wheat crop for two consecutive cropping seasons (2019–2020) at the Extension and Research Farm of School of Engineering, São Paulo State University (UNESP) at Selvíria, State of Mato Grosso do Sul, Brazil. The experimental field is located at geographical coordinates of 20°22′ S latitude, 51°22′ W longitude, and an altitude of 335 m (**Figure 1**). The climate of the region was classified as Aw type (humid tropical with a dry winter and rainy summer) as per the classification of Köppen-Geiger. The daily rainfall and temperature data in both cropping seasons of wheat are summarized in **Figure 2**. The soil was classified as Dystrophic Rhodic Haplustox with a clayey texture (Soil Survey Staff, 2014). The experimental area was grown with annual cereal and legume crops for over 28 years, while for the past 13 years, the area is under a no-tillage system (Santos et al., 2018).

Before beginning the experiment, 20 randomly selected soil samples were collected from the location of the experiment using a cup-type auger at a soil depth of 0.0–0.20 m. The soil samples were mixed to make a composite sample and analyzed according to the procedure of Raij et al. (2001) for the following chemical properties: pH (CaCl_2_) = 5.2; P (resin) = 37 mg dm^−3^; S-SO_4_^−2 =^ 5 mg dm^−3^, K^+^ = 2.3 mmol_c_ dm^−3^; Ca^2+^= 27 mmol_c_ dm^−3^; Mg^2+^= 17 mmol_c_ dm^−3^; Al^3+^= 0.01 mmol_c_ dm^−3^; H+Al = 35 mmol_c_ dm^−3^; cation exchange capacity = 69.1 mmol_c_ dm^−3^; base saturation = 52%; organic matter = 19 g kg^−1^; B (hot water) 0.15 mg dm^−3^. The pre-experiment soil analysis showed that diethylenetriaminepentaacetic acid (DTPA) extractable Zn content (0.7 mg dm^−3^) was medium low, while Fe = 29 mg dm^−3^, Mn = 41 mg dm^−3^, and Cu = 4 mg dm^−3^. A pre-experiment composite soil sample was also determined for the following granulometric attributes: clay (439 g kg^−1^), sand (471 g kg^−1^), and slit (90 g kg^−1^) as per the methodology of Teixeira et al. (2017).

### 2.2 Experimental design and treatments

Experiments were carried out in a completely randomized block design with four replications, arranged in a 4 × 5 factorial scheme. The treatments consisting of four types of seed inoculations (no inoculation, *A. brasilense, B. subtilis*, and *P. fluorescens*) and five nano-Zn foliar doses (0, 0.75, 1.5, 3.0, and 6.0 kg Zn ha^−1^) were applied 50% during tillering and 50% at the grain filling stage of wheat (Dhaliwal et al., 2019).

Seeds were manually inoculated in an individual plastic bag by mixing seeds with inoculant an hour before planting. Inoculation *via* seeds was performed using inoculants provided by the manufacturer (Biotrop^®^, Curitiba, Brazil). These inoculants are being commercially registered with the Ministry of Agriculture of Brazil with trade names of AzoTotal™ (*A. brasilense*), Vult™ (*B. subtilis*), and Audax™ (*P. fluorescens*). Wheat seeds were inoculated with *A. brasilense* strains Ab-V5 (CNPSo 2083) and Ab-V6 (CNPSo 2084) with a colony-forming unit (CFU) of 2 × 10^8^ ml^−1^ at a dose of 200 ml of liquid inoculant per 24 kg of seeds. The genome sequences of *A. brasilense* described that both strains Ab-V5 and Ab-V6 carried *fix* and *nif* genes that could promote nutrient transportation, biological nitrogen fixation, and the production of phytohormones, and invigorate tolerance against abiotic stresses (Fukami et al., 2018; Hungria et al., 2018). Inoculations with a *B. subtilis* strain (CCTB04) at 1 × 10^8^ CFU ml^−1^ and a *P. fluorescens* strain (CCTB03) at 2 × 10^8^ CFU ml^−1^ were performed at a dose of 150 ml ha^−1^ per 24 kg of wheat seeds. *B. subtilis* is carrying a non-ribosomal peptide synthetase and beta-glucanase to prevent phyto-pathogen infestation, helping in the bioremediation of heavy metal and Zn transporter (*zntR*) that could promote plant growth (Chaoprasid et al., 2015; Rekha et al., 2017; Muñoz-Moreno et al., 2018). *P. fluorescens* produces antibiotics, gluconic acid, and volatile organic compounds to deter soil pathogens, solubilize nutrients, and help in biological N fixation (David et al., 2018; Jing et al., 2020).

Foliar application of nano-Zn was performed from a liquid source of Zn (Nano R1 Zinco™), obtained from Allplant^®^ fertilizer industry, São Paulo, Brazil. The product is already registered with the Ministry of Agriculture, Brazil. Nano R1 zinc is characterized as a fluid suspension with 50% p/p Zn, 1,000 g L^−1^ solubility, 2.0 density, and 150-nm particle size, and is successfully used in previous studies to increase grain and plant Zn concentration (Nakao et al., 2018; Jalal et al., 2022c). Foliar application of nano-Zn was performed through a manual sprayer pump with a water capacity of 6.0 L (300 L ha^−1^ of volume application). Each foliar dose of nano-Zn was applied 50% during tillering and 50% at the grain filling stage of wheat (Dhaliwal et al., 2019). Spraying was carried out every morning. The field was visited soon after foliar spraying but no leaf damage was observed.

### 2.3 Plant materials

The experimental site was sprayed with herbicides [carfentrazone (40 g ha^−1^), glyphosate (1,800 g ha^−1^), and cletodim (240 g ha^−1^) of active ingredient (a.i.)] approximately 15 days prior to experimental initiation to control weeds with narrow and broad leaves. Wheat seeds were chemically treated with Standak Top^®^ [co-formulation of fungicides {thiram + carbendazim (105 g + 45 g of a.i.)} and insecticides {thiodicarb + imidacloprid (135 g + 45 g of a.i.)}] 100 kg^−1^ seeds, prior to inoculation and planting. Previous research reported that chemical treatment of cereal seeds before planting is a common agricultural practice in Brazil that could prevent infestation of soil pathogen without any drastic effect on seed inoculation of the same nature as the current experiment (Cardillo et al., 2019; Galindo et al., 2021; Jalal et al., 2023).

Wheat genotype (TBIO SOSSEGO) of potential production and quality was planted in a no-tillage system on 11 May 2019 and 3 May 2020 in the first and second cropping seasons, respectively. Sowing was carried out with a drill sowing method at 80 seeds m^−1^ while seedlings emerged approximately 5 days after planting. A basal dose of 270 kg ha^−1^ was applied from 08-28-16 (32, 112 and 64 kg ha^−1^ of N, P_2_O_5_, and K_2_O) at sowing on the basis of pre-experiment soil analysis and the recommendation of Boletim-100 for wheat crop (Cantarella et al., 1997). Each plot was composed of 13 rows, 5.0 m long and 0.17 m apart, for a total of 12.15 m^2^. The recommended dose of 120 N kg ha^−1^ was manually applied using the fertilizer ammonium sulfate during tillering (decimal growth stage-GS21) (Zadoks et al., 1974). Crops were irrigated with a sprinkler central-pivot irrigation system (14 mm on average) on the same day as fertilizer application to achieve uniform distribution and incorporation in all treatments. The experimental area had boron (B) deficiency (Vale et al., 2008). Therefore, all the treatments were homogeneously applied with 1.0 kg ha^−1^ of B from the source of boric acid (18% of B) through a tractor sprayer machine on the basis of pre-experiment soil analysis and interpretation of Campinas Agronomic Institute- IAC (Raij et al., 2001). The wheat crop was manually harvested on 12 September 2019 (with a 125-day cycle) and 7 September 2020 (with a 128-day cycle).

### 2.4 Evaluation and analysis

#### 2.4.1 Nutritional analysis

The plant materials (shoot and grain) were collected at physiological maturity in properly labeled paper bags and dried in an airtight oven at 60 ± 5°C for 72 h to measure nutritional analysis. The samples were ground in a stainless-steel Wiley knife mill by passing through a 10-mm-mesh sieve and stored in labeled plastic bags. Each sample was weighed (0.25 g), digested with nitroperchloric acid (HNO_3_:HClO_4_ solution), and quantified by atomic absorption spectrophotometry. The analysis was developed by following the methodology of Malavolta et al. (1997).

#### 2.4.2 Growth and productivity attributes

Plant height at physiological maturity was manually measured with a meter rod from the ground surface to the upper apex of the plant. Shoot dry matter was determined after harvesting from four central lines. The plants harvested from four central lines and samples were kept in an oven for 72 h at 60 ± 5°C to measure shoot dry matter. Wheat spikes were harvested from five central rows of 5-m length in bags and desiccated in the shade for approximately 7 days. An electric thresher was used for threshing individual samples and processed grains were weighed for conversion into grain yield per hectare at 13% moisture content.

#### 2.4.3 Zinc partitioning index, intake, and use efficiencies

The zinc partitioning index (ZPI) was derived from the fraction of grain-to-shoot Zn concentration following the methodology of Rengel and Graham (1996). Estimated daily Zn intake in Brazil with consumption of wheat grains on a daily basis was derived from the biofortified wheat grains in the present study, following the small modification in the study of Lessa et al. (2019). The consumption of wheat according to the Foreign Agricultural Service - United States Department of Agriculture (USDA. Foreign Agriculture Services, 2020) was 56.86 kg person^−1^ per annum (156 g person^−1^ per day) in Brazil. Following this information, the daily intake of biofortified wheat grains was calculated below in Eq. 1.


(Eq. 1) Zn intake=[Grain Zn]×C

where Zn grain (g kg^−1^) is the Zn concentration in biofortified grains in the present results and C (g person^−1^ per day) is the mean grain consumption of wheat per capita per day in Brazil.

Zinc use efficiency (ZnUE) and recovery applied Zn (RAZn) were calculated from the fraction of shoot Zn uptake to shoot dry matter and grain Zn uptake to grain yield using the procedure of Fageria et al. (2009); Fageria et al. (2011), and Jalal et al. (2021).


(Eq. 2) $$\text{ZnUE}=\frac{\text{GY ZnF }-\text{ GY ZnWF}}{\text{Zn applied dose }\left(\text{foliar}\right)}$$

(Eq. 3) $$\text{RAZn }\left(\%\right)=\frac{\text{ZnAF }-\text{ ZnAWF}}{\text{Zn applied dose }\left(\text{foliar}\right)}$$

where GY ZnF = grain yield with Zn fertilization, GY ZnWF = grain yield without Zn fertilization, ZnAF = shoot + grain Zn accumulation in Zn fertilized plots, and ZnAWF = shoot + grain Zn accumulation in Zn-fertilized plots.

### 2.5 Statistical analysis

All data were initially tested for normality using Shapiro and Wilk test, which showed that data are normally distributed (W ≥ 0.90). The data were submitted to analysis of variance (*F* test). Inoculations with PGPBs, Zn foliar doses, and their interactions were considered fixed effects in the model. When a main effect or interaction was significant by *F* test (*p* ≤ 0.05), then Tukey test (*p* ≤ 0.05) was used for the comparison of means of inoculations with PGPBs. In addition, regression analysis was performed for Zn foliar doses using R software (R Core Team, 2015).

The Pearson correlation analysis (*p* ≤ 0.05) was performed using R software (R Development Core Team). To create a heatmap, the package of corrplot with “cor” and “cor.mtest” functions was used to calculate the coefficients and *p*-value matrices. The digits added to the heatmap cells identify the significant correlations.

## 3 Results

### 3.1 Zinc, nitrogen, and phosphorus concentrations in wheat plant and grains

**Figure 3:**
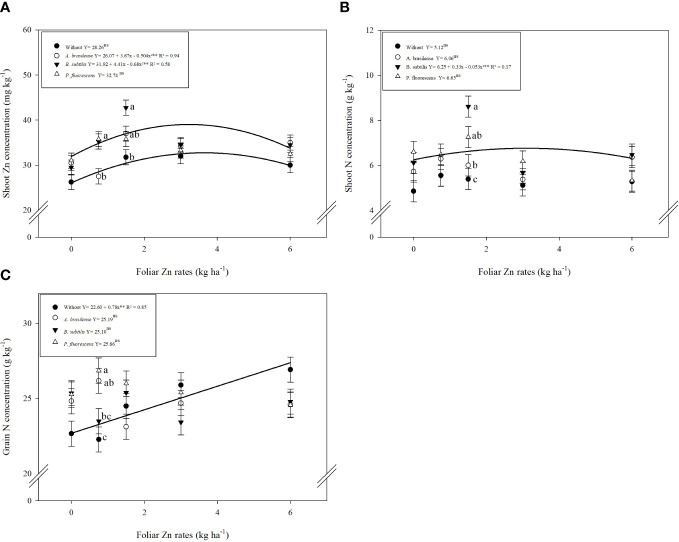
Influence of inoculation with PGPBs and nano-Zn foliar application on shoot Zn concentration in 2019 **(A)**, shoot N concentration in 2020 **(B)**, and grain N concentration in 2020 **(C)** of wheat. The different letters correspond to a significant difference at 5% probability level (*p* ≤ 0.05). The identical letters do not differ from each other as analyzed by Tukey test (PGPBs inoculations; *p* ≤ 0.05) and Regression (Foliar Zn rates; *p* ≤ 0.05) tests for wheat cropping year 2019 and 2020, respectively. Error bars indicate the standard error of the mean (*n* = 4 replications). Selvíria, 2020. **: significant at *p* ≤ 0.01.

The concentrations of zinc (Zn), nitrogen (N), and phosphorus (P) in shoot and grains of wheat were significantly influenced by inoculation with PGPBs and nano-Zn spray. Inoculation with PGPBs and foliar fertilization with nano-Zn significantly enhanced shoot Zn concentration of wheat in the cropping seasons of 2019 and 2020 (**Table 1**). The interaction for shoot Zn concentration was only significant in the 2019 cropping season (**Table 1**). The graphical trend in the 2019 cropping season indicated that increasing nano-Zn foliar spray up to 3.6 and 3.2 kg ha^−1^ in combination with inoculation of *A. brasilense* and *B. subtilis* increased shoot Zn concentration, respectively, while further increase in foliar nano-Zn fertilization led to the reduction of shoot Zn concentration. The combination of inoculation with *P. fluorescens* and foliar nano-Zn application was observed to be non-significant (**Figure 3A**). In the 2020 wheat cropping season, shoot Zn concentration was increased by 18.5% with inoculation of *P. fluorescens*, which was statistically similar to the inoculation of *B. subtilis* and *A. brasilense* as compared to without inoculation treatments (**Table 1**). The quadratic equation of foliar nano-Zn application was adjusted to 3.38 kg ha^−1^ to increase shoot Zn concentration in the 2020 cropping season (**Supplementary Table 1**).

**Table 1:** Shoot zinc (Zn), nitrogen (N), and phosphorus (P) concentrations of wheat as influenced by plant growth-promoting bacteria and foliar applied nano-Zn doses.

Treatments	Shoot Zn concentration		Shoot N concentration		Shoot P concentration	
	———————————— g kg^−1^ ————————————					
	2019	2020	2019	2020	2019	2020
Inoculations						
Without	29.5	32.9 b	5.4 b	5.2 b	1.17 b	1.15 b
*A. brasilense*	32.8	35.9 ab	5.5 b	5.9 ab	1.27 ab	1.27 ab
*B. subtilis*	35.3	37.1 a	6.7 a	6.4 a	1.26 ab	1.40 ab
*P. fluorescens*	33.6	39.0 a	6.3 ab	6.3 a	1.48 a	1.52 a
Foliar Zn application (kg ha^−1^)						
0	29.3	32.4	5.7	5.8	1.12	1.12
0.75	31.5	35.5	6.7	5.9	1.18	1.20
1.5	36.8	40.1	5.9	6.8	1.49	1.42
3	33.5	37.0	5.7	5.5	1.40	1.44
6	33.0	35.9	5.7	5.8	1.28	1.48
*F*-values						
Inoculation (I)	10.5**	8.9**	6.8**	7.2**	3.5*	5.5**
Foliar Zn (FZn)	10.8**	8.5**	2.9*	4.0**	3.7*	4.5**
I × FZn	2.06*	0.8 ns	0.9 ns	2.1*	1.7 ns	1.0 ns
**CV (%)**	10.23	10.5	16.7	15.6	23.9	22.7

Means in the column followed by different letters are significantly different (p-value ≤ 0.05); ** and * denote significant at p ≤ 0.01 and p ≤ 0.05, respectively, while ns indicates non-significant by F-test.

Shoot N concentration of wheat was positively increased with inoculation and foliar fertilization with nano-Zn in both cropping seasons (**Table 1**). Interaction of shoot N concentration was not significant in 2019 (**Supplementary Table 1**) and significant in the 2020 wheat cropping season (**Figure 3B**). Among inoculations, inoculation with *B. subtilis* enhanced shoot N concentration by 24% as compared to control treatments in first cropping season. Foliar application of nano-Zn at a dose of 1.5 kg ha^−1^ was observed with the highest shoot N concentration in the 2019 cropping season (**Table 1**). The shoot N concentration was adjusted to quadratic trend in the 2020 cropping season (**Figure 3B**). Shoot N concentration was enhanced with increasing foliar nano-Zn fertilization up to 3.11 kg ha^−1^ in combination with inoculation of *B. subtilis* while further increase in foliar nano-Zn doses led to the reduction of shoot N concentration of wheat (**Figure 3B**). In addition, the interactions of nano-Zn foliar doses with other inoculations were found to be non-significant (**Figure 3B**).

The interactions of inoculation with PGPBs and foliar fertilization with nano-Zn fertilization for shoot P concentration were not significant (**Table 1**). In addition, shoot P concentration of wheat was significantly enhanced with inoculation and foliar nano-Zn fertilization in the 2019 and 2020 cropping seasons (**Table 1**). Inoculation with *P. fluorescens* enhanced shoot concentration of P by 26.5% and 32.2% in the 2019 and 2020 cropping seasons, respectively, in comparison to the treatments without inoculation. The regression of foliar nano-Zn doses was adjusted to quadratic trend, where shoot P concentration was increased with a maximum estimated foliar nano-Zn dose of 5.0 and 4.4 kg ha^−1^ in 2019 and 2020, respectively (**Supplementary Table 1**). A further increase in foliar nano-Zn fertilization may cause reduction in shoot P concentration in the 2019 and 2020 cropping seasons.

The grain Zn concentration of wheat was positively influenced by inoculation and foliar nano-Zn fertilization in the 2019 and 2020 cropping seasons while their interactions were not significant in both studied cropping seasons (**Table 2**). Inoculation with *P. fluorescens* increased grain Zn concentration by 23.7% and 16.5%, which was statistically at par with inoculation of *B. subtilis* in 2019, and with inoculation of *B. subtilis* and *A. brasilense* as compared to without inoculation treatments in both seasons, respectively. The regression equation of nano-Zn foliar application was adjusted to a maximum estimated dose of 3.5 and 3.4 kg ha^−1^ for the higher grain Zn concentration of wheat in the 2019 and 2020 cropping seasons (**Supplementary Table 1**). These calculations indicated that a further increase of foliar nano-Zn fertilization may cause reduction in the grain Zn concentration of wheat.

**Table 2:** Grain zinc (Zn), nitrogen (N), and phosphorus (P) concentrations of wheat as a function of plant growth-promoting bacteria and foliar applied nano-Zn doses.

Treatments	Grain Zn concentration		Grain N concentration		Grain P concentration	
	——– mg kg^−1^ ——–		———————— g kg^−1^ ————————			
	2019	2020	2019	2020	2019	2020
Inoculations						
Without	37.6 c	44.2 b	24.5	24.4	2.49 b	2.53 c
*A. brasilense*	42.6 b	49.6 a	24.6	24.7	2.79 b	2.82 cb
*B. subtilis*	45.6 ab	50.8 a	25.3	24.5	3.16 a	3.15 a
*P. fluorescens*	46.5 a	51.5 a	24.8	25.6	2.79 b	2.91 ab
Foliar Zn application (kg ha^−1^)						
0	40.3	45.5	23.5	24.5	2.68	2.63
0.75	41.8	47.0	24.3	24.7	2.69	2.91
1.5	45.9	54.0	26.1	24.7	3.16	3.05
3	43.8	50.0	25.3	24.8	2.74	2.96
6	43.3	48.6	24.9	25.2	2.77	2.71
*F*-values						
Inoculation (I)	16**	7.2**	0.7 ns	2.1 ns	9.1**	8.8**
Foliar Zn (FZn)	3.6*	5.7**	3.8*	0.3 ns	3.7*	3.3*
I × FZn	1.3 ns	0.9 ns	1.1 ns	3.1**	1.6 ns	1.0 ns
**CV (%)**	10.2	11.2	7.87	6.7	14.4	13.5

Means in the column followed by different letters are significantly different (p-value ≤ 0.05); ** and * denote significant at p ≤ 0.01 and p ≤ 0.05, respectively, while ns indicates non-significant by F-test.

Grain N concentration was not significantly enhanced by inoculations or the interaction of foliar nano-Zn × inoculations in 2019, while the interaction in 2020 was significant (**Table 2**). The regression of foliar nano-Zn application was adjusted to a quadratic equation with a maximum estimated dose of 3.5 kg ha^−1^ for the increasing grain N concentration in the 2019 cropping season (**Supplementary Table 1**). In addition, grain N concentration was linearly enhanced with increasing foliar nano-Zn fertilization regardless of the inoculation in the 2020 cropping season of wheat as indicated in the graph trend (**Figure 3C**).

Inoculation and foliar nano-Zn doses positively enhanced grain P concentration while their interactions were not significant in both the 2019 and 2020 cropping seasons (**Table 2**). Grain P concentration was increased by 26.9% and 24.5% with the inoculation of *B. subtilis* as compared to without inoculation in the first and second cropping seasons, respectively. The regression analysis indicated that foliar nano-Zn was adjusted to a quadratic equation, where increasing foliar nano-Zn doses up to 3.0 and 3.4 kg ha^−1^ could increase grain P concentration while a further increase may cause reduction in grain P concentration of wheat in 2019 and 2020, respectively (**Supplementary Table 1**).

### 3.2 Shoot dry matter, yield, and zinc partitioning

Shoot dry matter of wheat was significantly increased by inoculation with PGPBs and foliar fertilization of nano-Zn while the interactions in both 2019 and 2020 cropping seasons were not significant (**Table 3**). Shoot dry matter was increased by 5.3% with the inoculation of *P. fluorescens* in both cropping seasons, which was statistically similar to treatments with the inoculation of *B. subtilis* and *A. brasilense* as compared to without inoculation. The regression analysis of nano-Zn foliar application was adjusted to a quadratic equation with a maximum Zn dose of 3.0 and 3.2 kg ha^−1^ in the 2019 and 2020 cropping seasons, respectively (**Supplementary Table 1**). Any further increase in nano-Zn foliar application may lead to the reduction of wheat shoot dry matter.

**Table 3:** Shoot dry matter, grain yield, and Zn partitioning index of wheat grains as a function of plant growth-promoting bacteria and foliar applied nano-Zn doses.

Treatments	Shoot dry matter		Grain yield		Zn partitioning index	
	——————– kg ha^−1^ ——————				—— % ——	
	2019	2020	2019	2020	2019	2020
Inoculations						
Without	4,843 b	4,909 b	3,436	3,341	70 b	131
*A. brasilense*	5,009 ab	5,095 a	3,508	3,705	75 b	139
*B. subtilis*	5,072 a	5,066 ab	3,582	3,731	78 ab	137
*P. fluorescens*	5,099 a	5,172 a	3,595	3,883	84 a	132
Foliar Zn application (kg ha^−1^)						
0	4,941	4,940	3,424	3,602	73	121
0.75	4,998	5,049	3,462	3,680	78	149
1.5	5,090	5,203	3,595	3,787	80	138
3	5,044	5,070	3,601	3,641	77	133
6	4,957	5,041	3,569	3,615	75	135
*F*-values						
Inoculation (I)	6.5**	6.4**	1.4 ns	17**	6.2**	0.9 ns
Foliar Zn (FZ)	1.5 ns	3.7**	1.3 ns	1.4 ns	1.0 ns	5.1**
I × FZ	1.5 ns	0.8 ns	2.8**	2.9 **	0.8 ns	6.4**
**CV (%)**	4	3.8	7.8	6.7	13.2	13.2

Means in the column followed by different letters are significantly different (p-value ≤ 0.05); ** and * denote significant at p ≤ 0.01 and p ≤ 0.05, respectively; ns indicates non-significant, by F-test.

**Figure 4:**
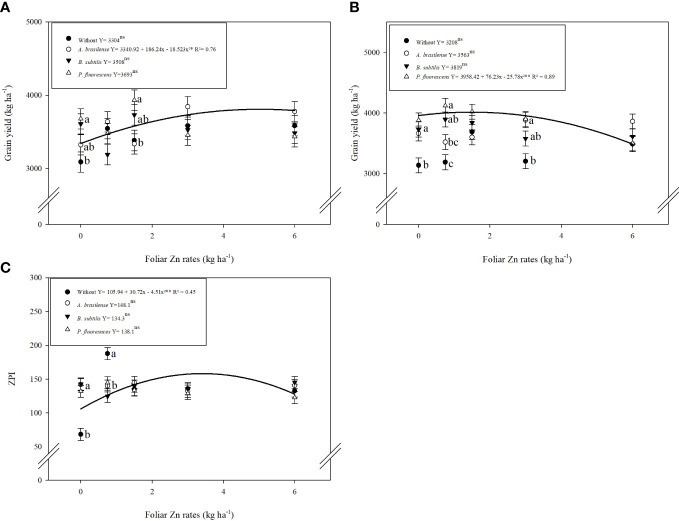
Influence of inoculation with PGPBs and nano-Zn foliar application on grain yield in 2019 **(A)** and 2020 **(B)**, and Zn partitioning index (ZPI) in 2020 **(C)** of wheat. The different letters correspond to a significant difference at 5% probability level (*p* ≤ 0.05). The similar letters of different means showed no difference as analyzed by Tukey test (PGPBs inoculations; *p* ≤ 0.05) and Regression (Nano-Zn foliar doses; *p* ≤ 0.05) tests for the first and second wheat cropping year, respectively. Error bars indicate the standard error of the mean (*n* = 4 replications). Selvíria, 2020. **: significant at *p* ≤ 0.01.

The interactive effect of inoculation with PGPBs and foliar nano-Zn doses positively enhanced the grain yield of wheat in the first and second cropping seasons (**Table 3**). The graph trend of the interaction of inoculations and nano-Zn foliar doses for grain yield of wheat in the 2019 and 2020 cropping seasons was set to a quadratic function (**Figures 4A, B**). Increasing foliar application of nano-Zn to 5 kg ha^−1^ in combination with inoculation of *A. brasilense* increased grain yield in the 2019 cropping season (**Figure 4A**). In addition, a maximum calculated dose of foliar nano-Zn up to 1.5 kg ha^−1^ in combination with the inoculation of *P. fluorescens* increased grain yield of wheat in the 2020 cropping season (**Figure 4B**). A further increase in foliar application of nano-Zn fertilization after calculated doses in the presence of inoculation with *A. brasilense* and *P. fluorescens* caused reduction in grain yield of wheat.

ZPI improved with the inoculation and application of foliar nano-Zn doses (**Table 3**). The interactive effect of foliar nano-Zn application and inoculations for ZPI was not significant in 2019 whereas interaction for ZPI was significant in the 2020 cropping season. ZPI was increased by 20% with the inoculation of *P. fluorescens* (**Table 3**). Foliar nano-Zn doses adjusted to a quadratic trend with an increasing dose of up to 3 kg ha^−1^ increased ZPI in wheat grain (**Supplementary Table 1**). The interactive effect of inoculation and foliar nano-Zn doses for ZPI in the 2020 cropping season was also adjusted to a quadratic trend (**Figure 4C**). The graph trend of foliar nano-Zn doses indicated that increasing nano-Zn application by up to 3.4 kg ha^−1^ regardless of inoculation increased ZPI in wheat grains (**Figure 4C**).

### 3.3 Zinc intake and Zn use efficiencies

The interactions of inoculation and nano-Zn foliar fertilization were not significant for the daily Zn intake with wheat consumption in both cropping seasons (**Supplementary Table 2**). Inoculation with *P. fluorescens* was observed with the maximum daily Zn intake from consumption of wheat in Brazil. Zinc intake was increased by 24% and 16% with the inoculation of *P. fluorescens* as compared to without inoculation in the 2019 and 2020 cropping seasons. The quadratic function for Zn intake described that increasing foliar nano-Zn application by up to 3.9 and 3.6 kg ha^−1^ improved the estimated daily Zn intake with wheat consumption in Brazil in the 2019 and 2020 cropping seasons, respectively (**Supplementary Table 1**). In both cases, a further increase in Zn-foliar doses may reduce estimated daily Zn intake in wheat grains.

**Figure 5:**
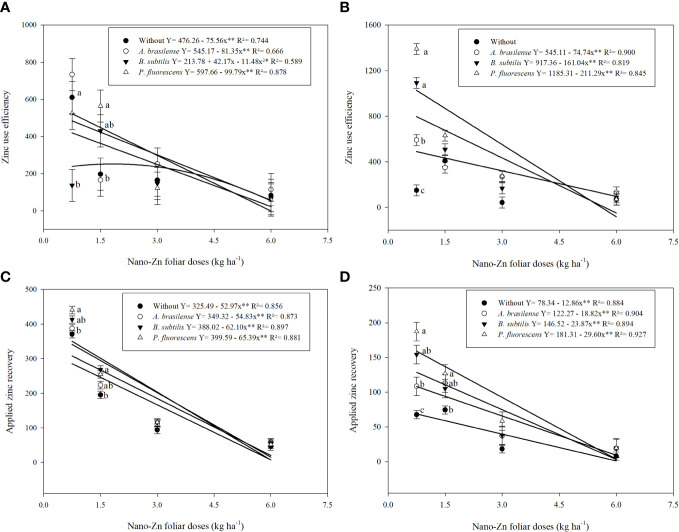
Influence of inoculation with PGPBs and nano-Zn foliar application on zinc use efficiency in 2019 **(A)** and 2020 **(B)**, and applied zinc recovery in 2019 **(C)** and 2020 **(D)** of wheat. The different letters correspond to a significant difference at 5% probability level (p ≤ 0.05). The identical letters do not differ from each other as analyzed by Tukey test (PGPBs inoculations; p ≤ 0.05) and Regression (Foliar Zn rates; p ≤ 0.05) tests for wheat cropping year 2019 and 2020, respectively. Error bars represent the standard error of the mean (n = 4 replications). ** significant at p ≤ 0.01.

The interactions between inoculations with PGPBs and foliar nano-Zn doses were significant for both Zn use efficiency (ZnUE) and applied Zn recovery (AZnR) (**Supplementary Table 2**). The graph trend indicated that ZnUE was linearly decreased with the inoculation of *A. brasilense* and *P. fluorescens* under increasing doses of nano-Zn foliar application in the first wheat cropping season. Zinc use efficiency with inoculation of *B. subtilis* was set to a quadratic trend with an increasing dose (1.8 kg ha^−1^) of nano-Zn foliar application while a further increase may cause reduction under the same inoculation (**Figure 5A**). In 2020, ZnUE was also linearly decreased with increasing doses of foliar nano-Zn application along with the inoculation of *A. brasilense, B. subtilis*, and *P. fluorescens*, while the interaction of foliar nano-Zn doses and without inoculation was not significant (**Figure 5B**). In addition, applied Zn recovery was linearly decreased with increasing foliar nano-Zn doses in 2019 (**Figure 5C**) and 2020 (**Figure 5D**), regardless of inoculation.

### 3.4 Pearson’s linear correlation

**Figure 6:**
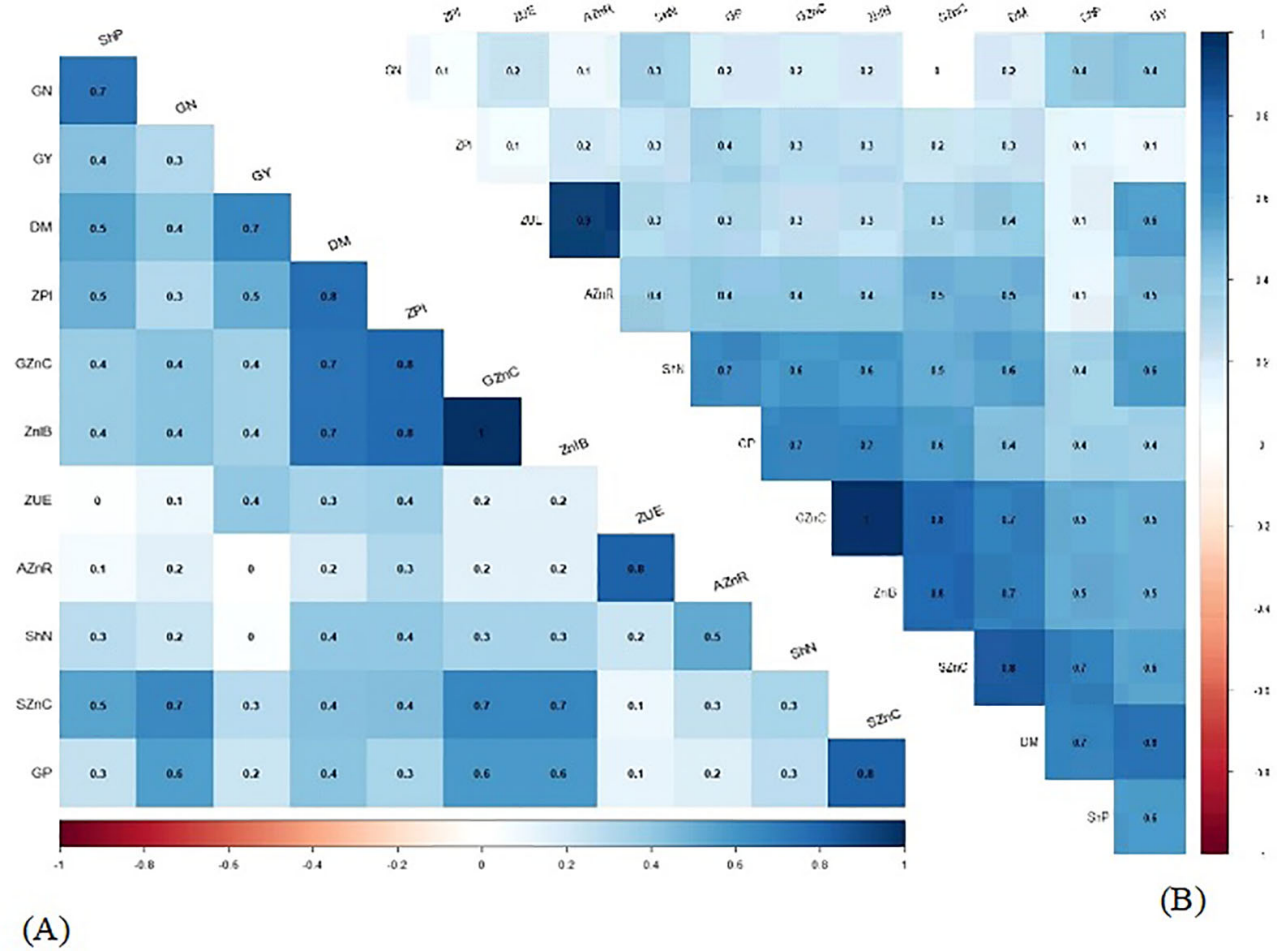
Heatmap color scale represented Pearson’s correlation between the attributes of wheat in the function of plant growth-promoting bacteria and foliar nano ZnO applications in 2019 **(A)** and 2020 **(B)** cropping seasons. DM, shoot dry matter; ShN, shoot N concentration; ShP, shoot P concentration; SZnC, shoot Zn concentration; GN, grain N concentration; GP, grain P concentration; GZnC, grain Zn concentration; GY, grain yield; ZPI, zinc partitioning index; ZnIB, estimated Zn intake in Brazil; ZnUE, Zn use efficiency; and AZnR, applied Zn recovery.

Pearson’s linear correlations between most of the evaluated attributes of wheat were positive and significant in 2019 (**Figure 6A**) and 2020 (**Figure 6B**). There was a positive correlation between grain N concentration and all evaluated attributes except applied Zn recovery and Zn use efficiency, which was positive but not significant in wheat cultivation during the 2019 crop season (**Figure 6A**).

In addition, there was a positive correlation between grain N concentration and all the evaluated attributes of wheat, except shoot N concentration in the 2020 cropping season (**Figure 6B**).

## 4 Discussion

Wheat grains are inherently low in Zn, which may result in not meeting daily human Zn requirement, particularly in regions with Zn deficiency. Therefore, a potential and sustainable strategy is needed to improve grain Zn concentration for health purposes (Jalal et al., 2020a; Jalal et al., 2020b). The integrated use of Zn and PGPBs is one of the sustainable and easy adaptable strategies that can effectively eliminate plant and human Zn scarcity by improving nutrition and productivity of wheat (Jalal et al., 2020b; Jalal et al., 2022a; Jalal et al., 2023). In this sense, the current experiment revealed that nano-Zn foliar application in combination with inoculation of PGPBs has increased N, P, and Zn concentrations in shoots and grains of wheat under field conditions (**Tables 1**, **2**). This might be due to the involvement of PGPBs in different soils and various mechanisms such as the production of phyto-hormones and enzymes, carboxylation, and biological fixation of nitrogen, which could help in the solubilization and availability of nutrients to plants for better absorption (Mitter et al., 2017; Rehman et al., 2021; Yasmin et al., 2022). In particular, it was reported that inoculation with *Pseudomonas* sp. could increase root architecture, branching, and proliferation, which will help host plants by improving their biochemical attributes and nutrient bioavailability to make plants healthy and for them to withstand harsh environmental conditions (Abadi et al., 2021; Yasmin et al., 2022). In addition, Zn is an important component and co-factor of several enzymes, cell division, and elongation, which help the plants maintain different biochemical activities for better crop growth, better physiology, more biofortified grains, and greater yield (Ullah et al., 2019; Jalal et al., 2020a; Faizan et al., 2021). In this sense, the present study demonstrated that inoculation with *B. subtilis* and *P. fluorescens* in combination with foliar fertilization of nano Zn increased the concentration of N, P and Zn in the shoot and grains of wheat (**Tables 1**, **2**; **Figure 3**). The positive correlations between the treatments supported the hypothesis of the current study (**Figure 6**). Previous studies also supported our results in that inoculation with *A. brasilense, B. subtilis*, and *P. fluorescens* could promote nutrient uptake and plant growth of different cereals and sugarcane (Galindo et al., 2022b; Rosa et al., 2022).

The current study indicated that inoculation with *P. fluorescens* and *B. subtilis* in combination with foliar fertilization of nano-Zn enhanced shoot dry matter and grain yield of wheat (**Table 3**; **Figure 4**). It may be possible due to the role of these PGPBs in developing a root system and biomass of host plants that act as a gateway for better nutrient absorption for better plant performance and greater productivity (Moretti et al., 2020). Previously, inoculation with *Bacillus* sp. along with zinc oxide has been considered as one of the effective strategies to improve the different physiological and biochemical traits of maize, thus enhancing growth and grain yield with high nutritional values under field conditions (Jalal et al., 2023). Co-application of PBPBs and Zn increased Zn use efficiency under tropical soils that ultimately promoted plant growth and yield of the maize–wheat cropping system (Galindo et al., 2021). It was previously described that the combined use of PGPBs and nano-Zn can efficiently stimulate the defense system of plants by enhancing primary metabolites and photosystems, which may lead to greater plant growth and grain yield (Tanveer et al., 2022). In addition, Zn is one of the important nutrients for plant growth regulation, cell multiplication, and biochemical mechanisms; all these functions together lead to higher dry matter and yield production (Doolette et al., 2020).

Wheat expansion to tropical and marginal regions is one of the best options to achieve food security; however, its inherent makeup of high ratio of phytic acid to Zn concentration in grains can cause malnutrition in Zn-deficient regions (Cakmak et al., 2010; Chattha et al., 2017). In this context, the current study indicated that foliar spray of nano-Zn along with the inoculation of *P. fluorescens* increased Zn partitioning and dietary intake of wheat grains (**Table 3**; **Figure 4C**). It has previously been stated that Zn-solubilizing bacteria such as *Bacillus* and *Pseudomonas* sp. strains stimulate several interactive mechanisms of soil and plant that convert the insoluble form of Zn into available Zn to enhance its uptake in plants for effective biofortification and higher yield (Hafeez et al., 2013; Singh et al., 2017; Jalal et al., 2021). Zinc-solubilizing bacteria could effectively increase bioavailability and assimilation of Zn in plants and grains by reducing phytic-P concentration, thus increasing these grains’ consumption by humans (Mumtaz et al., 2017). Some previous studies reported that the application of foliar Zn is highly mobile in phloem, and quickly assimilated and redistributed into new generating grains of wheat (Doolette et al., 2020; Rehman et al., 2021). The redistribution and re-localization of Zn into grain tissues of wheat could better handle Zn malnutrition in humans (Firdous et al., 2020).

Zinc efficiencies are better defined by the ratio of grain Zn concentration to Zn-deficient soils, where Zn use efficiencies could decrease with increasing Zn fertilization. Hence, the present study showed that inoculation with *P. fluorescens* in combination with nano-Zn foliar application increased Zn use efficiency and applied Zn recovery under field cultivation of wheat (**Figure 5**). This might be due to the effectiveness of PGPBs in the dissolution of oxides, sulfides, and carbonates, thus increasing Zn bioavailability for better use of plants (Anuradha et al., 2015). Previous studies performed by Ullah et al. (2020) and Jalal et al. (2022a); Jalal et al. (2022b) reported that inoculation with PGPBs along with Zn fertilization could increase Zn use efficiencies *via* cultivation of different crops on soils with a low Zn content.

## 5 Conclusion

The combined use of foliar nano-Zn and PGPBs is among the rapid and sustainable alternative strategies for cereal production. It was verified from our results that inoculation with PGPBs in combination with nano-Zn foliar application increased plant and grain N, P and Zn concentrations, growth and yield of wheat. The inoculation with *B. subtilis* and *P. fluorescens* enhanced concentrations of nitrogen, phosphorus, and Zn in shoot and grain as well as provided greater shoot dry matter, grain yield and ZPI in wheat. The inoculation of *B. subtilis* and *P. fluorescens* along with the optimal calculated dose of foliar nano-Zn fertilizer, ranging from 3 to 3.5 kg ha^-1^, increased most of the evaluated traits of wheat. Zinc intake from daily wheat consumption in Brazil, applied Zn recovery, and Zn use efficiency increased with the combined *P. fluorescens* and nano-Zn foliar application under field conditions. In this context, combined application of *P. fluorescens* and foliar nano-Zn under tropical savannah regions could be an effective strategy to improve plant nutrient acquisition and use efficiencies, particularly Zn, leading to sustainable production and biofortification of wheat. Prospective research should focus on the improvement of Zn use efficiency and recovery as well as the hormonal regulators produced as a result of inoculation and co-inoculation with PGPBs, and their influence on the performance, biofortification, and physiological traits of cereals to efficiently understand solubilization, assimilation, and partitioning of Zn under field conditions.

## Data availability statement

The datasets presented in this study can be found in online repositories. The names of the repository/repositories and accession number(s) can be found below: http://hdl.handle.net/11449/238501.

## Author contributions

Conceptualization and administration of the study: AJ and MF. Methodology, validation, and formal analysis: AJ, CO, GF and FG. Investigation, resources, and data curation: AJ, AB, KC, GL and JS. Writing—original draft preparation: AJ. Writing—review and editing: MF, ES and FG. Supervision, project administration, and funding acquisition: AJ and MF. All authors contributed to the article and approved the submitted version.
