# Supplementary material for: Integrated use of plant growth-promoting bacteria and nano-zinc foliar spray is a sustainable approach for wheat biofortification, yield, and zinc use efficiency
Source: Front Plant Sci. 2023 May 8;14:1146808. doi: 10.3389/fpls.2023.1146808 (PMC10200892; doi:10.3389/fpls.2023.1146808)
Supplement: Supplementary file 1 [file DataSheet_1.docx]

Supplementary Material

Integrated Use of Plant Growth-Promoting Bacteria and Nano-Zinc Foliar Spray is a Sustainable Approach for Wheat Biofortification, Yield and Zinc Use Efficiency

Arshad Jalal^1^, Carlos Eduardo da Silva Oliveira^1^, Guilherme Carlos Fernandes^1^, Edson Cabral da Silva^1^, Kaway Nunes da Costa^1^, Jeferson Silva de Souza^1^, Gabriel da Silva Leite^1^, Antonio Leonardo Campos Biagini^1^, Fernando Shintate Galindo^2^, Marcelo Carvalho Minhoto Teixeira Filho^1^*

^1^Department of Rural Engineering, Plant Health and Soils, São Paulo State University (UNESP), 15385-000, Ilha Solteira, SP, Brazil

^2^Faculty of Agricultural Sciences and Technology, Department of Plant Production, São Paulo State University (UNESP), Dracena, SP, Brazil

* Correspondence:

Marcelo Carvalho Minhoto Teixeira Filho

mcm.teixeira-filho@unesp.br

**Supplementary Table 1.** Concentrations of Zn, N, P in shoot and grains, shoot dry matter, Zn partitioning index and estimated daily Zn intake as a function of foliar nano-Zn application regardless of the inoculations in 2019 and 2020 cropping seasons of wheat.

| **Sources** | **Equations** |
| --- | --- |
| Shoot Zn concentration 2020 | y = 33.44+3.38x-0.50x^2^ (R^2^ = 0.58^ns^) |
| Shoot N concentration 2019 | y = 6.11-0.02x-0.0078^2^ (R^2^ = 0.15^ns^) |
| Shoot P concentration 2019 | y = 1.12+0.20x-0.02x^2^ (R^2^ = 0.70^**^) |
| Shoot P concentration 2020 | y = 1.12+0.17x-0.01x^2^ (R^2^ = 0.90^**^) |
| Grain Zn concentration 2019 | y = 40.77+2.56x-0.36x^2^ (R^2^ = 0.60^**^) |
| Grain Zn concentration 2020 | y = 45.95+3.74x-0.55x^2^ (R^2^ = 0.51^**^) |
| Grain N concentration 2019 | y = 23.72+1-22x-0.17x^2^ (R^2^ = 0.67^**^) |
| Grain P concentration 2019 | y = 2.72+1.12 x-0.02 x^2^ (R^2^ =0.17^ns^) |
| Grain P concentration 2020 | y = 2.70+0.23x-0.03x^2^ (R^2^ = 0.82^**^) |
| Shoot dry matter 2019 | y = 4952+81.86x-13.64x^2^ (R^2^ = 0.78^*^) |
| Shoot dry matter 2020 | y = 4979+104.1x-15.9x^2^ (R^2^ = 0.48^*^) |
| Zinc partitioning index 2019 | y = 75.48+2.73x-0.45x^2^ (R^2^ = 0.50^ns^) |
| Zinc intake (Brazil) 2019 | y = 6.36+0.39x-0.05x^2^ (R^2^ = 0.61^**^) |
| Zinc intake (Brazil) 2020 | y = 7.16+0.58x-0.08x^2^ (R^2^ = 0.51^**^) |

** Significant and ^ns^ = non-significant at p ≤ 0.01

# Supplementary Table 2. Zinc intake in Brazil, Zn use efficiency (ZnUE) and applied Zn recovery (AZnR) in wheat as influenced by plant growth-promoting bacteria and foliar applied nano-Zn doses.

| **Treatments** | Zn intake (Brazil) | | ZnUE | | AZnR | |
| --- | --- | --- | --- | --- | --- | --- |
|  | g person^−1^ day^−1^ | | kg kg^−1^ | | % | |
|  | 2019 | 2020 | 2019 | 2020 | 2019 | 2020 |
| **Inoculations** |  | |  | | | |
| Without | 5.8 c | 6.9 b | 263 | 167 c | 176 c | 42 c |
| *A. brasilense* | 6.6 b | 7.7 a | 316 | 335 bc | 195 cb | 69 b |
| *B. subtilis* | 7.1 ab | 7.9 a | 195 | 464 ab | 213 ab | 79 ab |
| *P. fluorescens* | 7.2 a | 8.0 a | 317 | 591 a | 216 a | 98 a |
| **Foliar Zn application (kg ha^−1^)** | | | | | | |
| 0 | 6.3 | 7.0 | ----- | ---- | ----- | ----- |
| 0.75 | 6.5 | 7.3 | 501 | 805 | 403 | 129 |
| 1.5 | 7.2 | 8.4 | 339 | 474 | 235 | 105 |
| 3 | 6.8 | 7.8 | 171 | 188 | 109 | 38 |
| 6 | 6.7 | 7.5 | 80 | 90 | 53 | 17 |
| **F values** |  |  |  |  |  |  |
| Inoculation (I) | 16** | 7.2** | 1.77ns | 13.5** | 11.6** | 12.4** |
| Foliar Zn (FZn) | 3.6* | 5.7** | 18.4** | 42.6** | 844** | 65** |
| I x FZn | 1.3 ns | 0.9 ns | 4.13** | 6.7** | 2.38* | 2.58* |
| **CV (%)** | 10.2 | 11.2 | 63.45 | 50.6 | 10.7 | 36.6 |

Means in the column followed by different letters are significantly different (*p*-value ≤ 0.05); ** and *—significant at p ≤ 0.01 and p ≤ 0.05, respectively; ns—non-significant, by F-test.
